# Factors That Could Impact on Liver Fibrosis Staging by Transient Elastography

**DOI:** 10.1155/2015/624596

**Published:** 2015-12-06

**Authors:** Hugo Perazzo, Valdilea G. Veloso, Beatriz Grinsztejn, Chris Hyde, Rodolfo Castro

**Affiliations:** ^1^Evandro Chagas National Institute of Infectious Disease (INI), Oswaldo Cruz Foundation (FIOCRUZ), Laboratory of Clinical Research on STD/AIDS, Avenida Brasil 4365, 21040-900 Manguinhos, RJ, Brazil; ^2^Institute of Health Research, Peninsula Technology Assessment Group (PenTAG), Evidence Synthesis and Modelling for Health Improvement (ESMI), University of Exeter Medical School, University of Exeter, St. Luke's Campus, South Cloisters, EX1 2LU Exeter, UK

## Abstract

Transient elastography (TE) based on liver stiffness measurement (LSM) is one of the most validated noninvasive methods for liver fibrosis staging in patients with chronic liver diseases. This method is painless, has no potential complications, is rapid (<10 min), and can be performed at the patient's bedside. However, several points should be considered when interpreting TE results. This review aims to discuss the critical points that might influence liver stiffness and TE results. Spectrum bias and the impact of the prevalence of fibrosis stages should be taken into account when interpreting the studies that validated this method using liver biopsy as a gold-standard. LSM might be influenced by nonfasting status, flare of transaminases, heart failure, extrahepatic cholestasis, presence of steatosis, aetiology of liver disease, type and position of probe, and operator's experience. In addition, interobserver variability can impact on the management of patients with chronic liver diseases. TE should be performed by an experienced operator (>100 exams), in a 3-hour fasting status, and its results should be handled by specialist clinicians that are aware of the limitations of this method.

## 1. Introduction

Transient elastography (TE) by Fibroscan (EchoSens, Paris, France) is one of the most widely used and validated noninvasive methods for liver fibrosis staging [1]. This method is painless, is not associated with potential complications, and is well accepted by patients, especially for repeated examinations [2]. TE is accurate for staging liver fibrosis [1] and can be used for prediction of mortality and severe outcome in patients with chronic liver diseases [3, 4]. However, several points should be considered when using TE for liver fibrosis staging to avoid misclassification of patients [5]. Some methodologic issues should be taken into account when interpreting studies that validate this method. Liver stiffness can be influenced by nonfasting status, flare of transaminases, cardiac congestion, and extrahepatic cholestasis. In addition, the type and position of probe and operator experience can also impact on TE results.

## 2. Technical Principles of Transient Elastography

A transducer probe transmits vibrations of mild amplitude and low frequency (50 Hz) inducing elastic shear waves that propagate through the liver tissue. Propagation of shear waves is followed using a pulse-echo ultrasound acquisition measuring its velocity that is directly correlated with liver stiffness: the stiffer the tissue is, the faster the shear wave propagates. The examination is rapid (less than 10 min) and can easily be performed at the patient's bedside. Briefly, the probe is placed in an intercostal space at the intersection between the xyphoid and the median axillary line (i.e., on the right lobe of the liver, usually where liver biopsy would be performed). The operator, assisted by a time-motion ultrasound image, locates a liver portion free of large vascular structures and acquires the measurement (“shot”). The software determines whether the liver stiffness measure (LSM) is valid or not; when a measure is invalid, it is automatically discharged. The result of the TE represents the median of all valid acquisitions and ranges from 2.5 to 75.0 kPa [6]. TE should be considered reliable when all the following criteria are met: (i) 10 successful measurements; (ii) an interquartile range lower than 30% of the median value; and (iii) a success rate of more than 60% [7]. In a study that evaluated more than 13,000 TE examinations, unreliable results were independently associated with body mass index, lower operator experience, older age, female gender, and metabolic factors [8].

Currently, two probes are available for staging of liver fibrosis in adults: (i) M probe, the standard probe used in patients with normal weight, and (ii) XL probe, used in obese patients or when TE results with M probe are unreliable. TE results have been described as unreliable in around 20% of cases using the M probe [8] and in lower rates within the XL probe [9]. In both probes liver stiffness is measured in volume such as a cylinder being 4 cm high and 1 cm wide (100 times higher than liver biopsy). Technical differences between the M and XL probes include their central ultrasound frequency (3.5 versus 2.5 MHz), vibration amplitude (2 versus 3 mm), and the diameter of their tips (9 versus 12 mm). In addition, measures from XL probe are deeper compared to those performed by M probe [9]. The “normal” values of TE were defined in healthy individuals as around 5.5 kPa with the M probe, showing that liver stiffness was higher in males compared to females and in obese individuals compared to those with normal weight [10, 11].

## 3. Critical Analysis of Diagnostic Performance

The standard expression of the effectiveness of a diagnostic test is represented by the area under the receiver operator characteristic curve (AUROC), which plots the sensitivity over 1−specificity. TE is an accurate tool for stage liver fibrosis with AUROC ranging from 0.79 to 0.83 for significant fibrosis (*F* ≥ 2) and from 0.95 to 0.97 cirrhosis (*F* = 4) in patients with chronic liver diseases [2]. In most studies AUROC curves for TE were calculated using liver biopsy as the gold standard. In this context, the result represents the probability that TE will correctly rank random patients as “significant fibrosis or cirrhosis” or as “no fibrosis” based on liver fibrosis [12]. This methodology leads to major issues: the assumption that liver biopsy is binary or dichotomy, whereas fibrosis staging uses a five-stage ordinal scale (from *F*0 to *F*4) and the impact of diagnostic performance of noninvasive tests based on the spectrum bias [13]. In addition, the diagnostic performance of TE might be influenced by liver biopsy limitations [14].

The spectrum bias and the impact of the prevalence of fibrosis stages in AUROC estimation were demonstrated in a study that used a serum noninvasive method (FibroTest). AUROC values ranged from 0.67 to 0.98 according to how “advanced fibrosis” was defined: the lowest AUROC value was observed when only stage *F*2 was defined as advanced fibrosis and only stage *F*1 as nonadvanced fibrosis (*F*1 versus *F*2) and the maximal value was observed when the authors evaluated *F*0 (defined as nonadvanced fibrosis) versus *F*4 (advanced fibrosis) [15]. The adjustment of AUROC values using the Obuchowski method is an alternative to overcome the spectrum bias and the ordinal classification of liver biopsy [16]. This methodology is a multinomial version of the AUROC, with *N* = 5 (i.e., *F*0, *F*1, *F*2, *F*3, and *F*4) categories of liver biopsy. Thus, the “weighted AUROC” (wAUROC) by the Obuchowski method is a weighted average of the *N*(*N* − 1)/2 different curves corresponding to all pairwise comparisons between 2 × 2 of the *N* categories. Each pairwise comparison is weighted according to the distance between fibrosis stages with a penalty function proportional to the difference in METAVIR classes between stages. This penalty function was defined as 0.25 per difference among fibrosis stages. When the differences between METAVIR classes are one (i.e., *F*0 to *F*1), two (*F*0 versus *F*2), three (*F*0 versus *F*3), or four stages (*F*0 versus *F*4), the penalty is 0.25, 0.50, 0.75, or 1.00, respectively.

The impact of the spectrum bias and the fact that liver biopsy is not dichotomy were validated in a study using TE for stage fibrosis in patients with chronic hepatitis C (CHC) and healthy volunteers (controls). Analyzing only patients, this study showed lower AUROC (95% CI) values between adjacent stages [*F*1 versus *F*2: AUROC = 0.613 (0.573–0.650)] than between aggregated stages [AUROC = 0.745 (0.716–0.771) for *F*0*F*1 versus *F*2*F*3*F*4 and AUROC = 0.852 (0.823–0.877) and *F*0*F*1*F*2*F*3 versus *F*4, resp.]. In addition, AUROC values for diagnosis of fibrosis stage ≥2 were higher when analyzing all individuals (patients and controls) compared to the analysis of CHC patients without controls [AUROC = 0.830 (0.810–0.848) versus 0.761 (0.734–0.785), *p* < 0.001]. This difference might be explained by the fact that authors changed the prevalence of fibrosis stages in the sample, increasing the proportion of individuals with *F*0 stage, by adding the controls. However, wAUROC (95% CI) were similar in these two analyses [wAUROC = 0.894 (0.887–0.901) versus 0.883 (0.874–0.892); *p* = NS] when analyzing CHC patients and controls versus CHC patients showing that the Obuchowski method is not influenced by this bias [17].

The diagnostic performance [AUROC (95% CI)] of TE with and without the Obuchowski method was recently reported in a study with a prevalence of fibrosis stages of 54%, 30%, 9%, and 7% for *F*0*F*1, *F*2, *F*3, and *F*4, respectively. AUROC values were 0.874 (0.811–0.937) for *F* ≥ 2 and 0.942 (0.890–0.993) for *F* = 4. The wAUROC was 0.89 (0.86–0.93) when the Obuchowski method was used to adjust the diagnostic performance of the noninvasive test [18]. In addition, similar TE diagnostic performance was reported in meta-analyses in patients with chronic hepatitis B [wAUROC = 0.89 (0.83–0.96)] [19] and chronic hepatitis C [0.88 (0.87–0.89)] [17] adjusted by the Obuchowski method.

## 4. Conditions That Lead to Overestimation of Liver Fibrosis by TE

### 4.1. Flare of Transaminases

Preliminary studies reported that severe necroinflammatory activity, such as alanine transaminases (ALT) greater than 10 times the upper limit of normal (ULN), might lead to an increase in liver stiffness and an overestimation of fibrosis estimation [20]. More recent studies showed that even in lesser grades of activity liver stiffness might be overestimated. In patients with chronic hepatitis B having the same fibrosis stage by liver biopsy, those with ALT levels ≥2 times ULN had higher TE results compared to those with normal transaminases (9.5 versus 4.7 kPa, *p* > 0.001) [21]. This hypothesis was validated by analyzing a significant decrease in liver stiffness values after a 3-month antiviral treatment in chronic hepatitis B compared to pretreatment levels (7.9 versus 6.4 kPa; *p* < 0.001) [22]. Patients had elevated transaminases that normalized after 3-month antiviral treatment and the time between first and second measures was a very short delay to consider regression of liver fibrosis. Similar results were reported in chronic hepatitis C: considering patients with fibrosis stages ≥*F*3 identified by liver biopsy, those with important necroinflammatory activity (*A*2*A*3*A*4) had higher liver stiffness measurements compared to those without activity (*A*0*A*1) [14.6 versus 6.2 kPa; *p* < 0.05] [23].

### 4.2. Extrahepatic Cholestasis and Liver Congestion

Liver stiffness values increase in extrahepatic cholestasis independently of fibrosis status. Studies reported that liver stiffness highly correlates with total bilirubin levels (Spearman's correlation *r* = 0.67 and 0.69) and decreases after successful biliary drainage (from 10.8 to 7.1 kPa and 7.6 to 5.4 kPa in both studies) [24, 25]. The reasons underlying the high stiffness in cholestasis are unknown but could be related to tissue swelling, inflammation, edema, and increased intracellular pressure due to impaired bile flow. In addition, the increased hydrostatic pressure alone seems to contribute to increased liver stiffness during extrahepatic cholestasis [24].

Hepatic congestion might increase liver stiffness measurements leading to an overestimation of fibrosis and a misclassification of cirrhosis. In a case-report, authors showed a significant decrease of liver stiffness after a cardiac transplant (44.3 versus 3.8 kPa; *p* < 0.0001) in a patient with chronic heart failure. This hypothesis was validated in few studies that evaluated TE in patients with cardiac dysfunction and controls. Liver stiffness measurements were significantly higher in patients with heart failure than in controls and decreased during hospitalization after control of cardiac disease [26]. In addition, liver stiffness was increased in patients with right-sided heart failure compared to healthy controls [9.7 (IQR 5.0–10.8) kPa versus 4.4 (3.6–5.1) kPa, *p* < 0.001] [27]. These findings can be explained by worsening cardiac dysfunction increasing hepatic vein pressure leading to intrahepatic blood stasis and higher liver stiffness.

### 4.3. Nonfasting Status

Liver stiffness measurement significantly increased immediately after food intake (6.0 ± 2.1 versus 4.9 ± 2.1 kPa; *p* < 0.001) in patients with no fibrosis and moderate fibrosis (8.2 ± 2.3 versus 7.2 ± 1.3 kPa; *p* = 0.008). In addition, TE results normalized 180 min after meal [28]. The impact of nonfasting status on liver stiffness was validated in patients with CHC: an increase in liver stiffness was observed 15 to 45 minutes after the onset of the meal with return to baseline premeal levels within 120 minutes [29]. These results reinforce that TE should be performed in 120 to 180 min fasting status.

### 4.4. Liver Steatosis

Liver steatosis might impact in TE leading to an overestimation of liver fibrosis. Liver stiffness values were significantly higher in subjects with severe steatosis (≥66% at liver biopsy) compared to those without (*F*0-*F*1 6.9 versus 5.8 kPa, *p*  =  0.04; *F*0–*F*2 7.4 versus 6.0 kPa, *p*  =  0.001) in a study with biopsy-proven patients with nonalcoholic fatty liver disease. In addition, a higher rate of false-positive TE results was observed in patients with steatosis ≥66% compared to those without (*F*0-*F*1 23.6% versus 14.9%, *F*0–*F*2 33.3% versus 13.2%) [30]. Similar results were observed in patients with CHC: amongst patients within the same fibrosis stages (*F*0–*F*2 and *F*3-*F*4; *F*0–*F*3 and *F*4), mean liver stiffness values were significantly higher in subjects with moderate-severe steatosis (≥20% at liver biopsy) compared to those without [31].

## 5. Other Factors That Might Impact on Transient Elastography Results

### 5.1. Operator Effect

TE reliability and its diagnostic performance might be influenced by the operator's experience. The experience of the operator was independently associated with unreliability of this method in a study that analyzed more than 13,000 examinations [8]. An operator effect in TE was validated in a study that used FibroTest as the reference: the diagnostic performance of TE was significantly better when examinations performed by the nonexperimented operator were excluded (AUROC = 0.80 versus 0.70; *p* = 0.009) [7]. The performance of at least 100 examinations should be considered to define an experienced operator [32].

### 5.2. Interobserver Variability

Controversial results have been described in recent years on reproducibility of TE. The first study reported a high intraclass correlation coefficient [ICC = 0.990 (95% CI 0.977–0.987)] [33]. Besides the description of similar results, other authors showed 25% of discordance of at least one stage of liver fibrosis by TE performed by experienced operators [34]. This relatively high rate of classification of fibrosis stages was reported in more recent studies [32, 35–37]. Both examinations were performed by an experienced operator, were well correlated, and presented high ICC values. However, these studies reported a rate from 20 to 30% discrepancy of at least one stage of fibrosis. We are aware that it is very difficult, even with liver biopsy, to distinguish between intermediate adjacent fibrosis stages (i.e.; *F*2 versus *F*3) [38]. However, this variability of at least one stage of fibrosis might impact on the management of patients with chronic liver disease. The correct classification of fibrosis stage might impact on access to new direct-acting antiviral (DAA) treatment for patients with CHC or duration of treatment [39]. This variability might be associated with the choice of intercostal space or probe position [40] or might be related to the TE technique itself and a few factors that cannot be controlled during examination. TE results performed in a longitudinal follow-up (repeated measurements) might be more accurate than a single measure and interobserver variability might be minimized when TE measures are performed by the same experienced operator during follow-up [41].

### 5.3. Other Factors

Liver stiffness might also be impacted by the aetiology of the chronic liver disease. Patients with cholestatic liver diseases, such as primary biliary cirrhosis and primary sclerosing cholangitis, seem to have higher stiffness than those with viral hepatitis. Therefore, for each stage of fibrosis, cutoffs are higher than in chronic viral hepatitis either because of the nature of the liver disease or because of cholestasis [2]. Similar higher cutoffs for each fibrosis stage were described in alcoholic liver disease [42]. There is no evidence that presence of hepatocellular carcinoma might impact on liver stiffness. However, TE has a prognostic value to predict the development of liver neoplasm [43].

## 6. Conclusions

Liver stiffness might be influenced by nonfasting status, flare of transaminases, heart failure, extrahepatic cholestasis, and presence of severe steatosis leading to an overestimation of liver fibrosis. In addition, a not negligible interobserver variability can also impact on management of patients with chronic liver diseases. The prevalence of fibrosis stages and the fact that liver biopsy is not dichotomy might have impacted on the diagnostic performance of the test. Transient elastography should be performed by an experienced operator (>100 exams), in a fasting status, and its results should be handled by specialist clinicians that are aware of limitations of this method.

## References

[1] Bota S., Herkner H., Sporea I. (2013). Meta-analysis: ARFI elastography versus transient elastography for the evaluation of liver fibrosis. *Liver International*.

[2] de Lédinghen V., Vergniol J. (2008). Transient elastography (FibroScan). *Gastroentérologie Clinique et Biologique*.

[3] Singh S., Fujii L. L., Murad M. H. (2013). Liver stiffness is associated with risk of decompensation, liver cancer, and death in patients with chronic liver diseases: a systematic review and meta-analysis. *Clinical Gastroenterology and Hepatology*.

[4] Snowdon V. K., Guha N., Fallowfield J. A. (2012). Noninvasive evaluation of portal hypertension: emerging tools and techniques. *International Journal of Hepatology*.

[5] Perazzo H., Fernandes F. F., Filho E. C. C., Perez R. M. (2015). Points to be considered when using transient elastography for diagnosis of portal hypertension according to the Baveno's VI consensus. *Journal of Hepatology*.

[6] Castera L., Forns X., Alberti A. (2008). Non-invasive evaluation of liver fibrosis using transient elastography. *Journal of Hepatology*.

[7] Poynard T., Ingiliz P., Elkrief L. (2008). Concordance in a world without a gold standard: a new non-invasive methodology for improving accuracy of fibrosis markers. *PLoS ONE*.

[8] Castera L., Foucher J., Bernard P.-H. (2010). Pitfalls of liver stiffness measurement: a 5-year prospective study of 13,369 examinations. *Hepatology*.

[9] de Lédinghen V., Wong V. W.-S., Vergniol J. (2012). Diagnosis of liver fibrosis and cirrhosis using liver stiffness measurement: comparison between M and XL probe of FibroScan. *Journal of Hepatology*.

[10] Roulot D., Czernichow S., Le Clésiau H., Costes J.-L., Vergnaud A.-C., Beaugrand M. (2008). Liver stiffness values in apparently healthy subjects: influence of gender and metabolic syndrome. *Journal of Hepatology*.

[11] Das K., Sarkar R., Ahmed S. M. (2012). ‘Normal’ liver stiffness measure (LSM) values are higher in both lean and obese individuals: a population-based study from a developing country. *Hepatology*.

[12] Hanley J. A., McNeil B. J. (1982). The meaning and use of the area under a receiver operating characteristic (ROC) curve. *Radiology*.

[13] Lambert J., Halfon P., Penaranda G., Bedossa P., Cacoub P., Carrat F. (2008). How to measure the diagnostic accuracy of noninvasive liver fibrosis indices: the area under the ROC curve revisited. *Clinical Chemistry*.

[14] Mehta S. H., Lau B., Afdhal N. H., Thomas D. L. (2009). Exceeding the limits of liver histology markers. *Journal of Hepatology*.

[15] Poynard T., Halfon P., Castera L. (2007). Standardization of ROC curve areas for diagnostic evaluation of liver fibrosis markers based on prevalences of fibrosis stages. *Clinical Chemistry*.

[16] Obuchowski N. A. (2006). An ROC-type measure of diagnostic accuracy when the gold standard is continuous-scale. *Statistics in Medicine*.

[17] Poynard T., de Ledinghen V., Zarski J.-P. (2011). FibroTest and Fibroscan performances revisited in patients with chronic hepatitis C. Impact of the spectrum effect and the applicability rate. *Clinics and Research in Hepatology and Gastroenterology*.

[18] Fernandes F. F., Ferraz M. L., Andrade L. E. (2015). Enhanced liver fibrosis panel as a predictor of liver fibrosis in chronic hepatitis C patients. *Journal of Clinical Gastroenterology*.

[19] Poynard T., Ngo Y., Munteanu M., Thabut D., Ratziu V. (2011). Noninvasive markers of hepatic fibrosis in chronic hepatitis B. *Current Hepatitis Reports*.

[20] Arena U., Vizzutti F., Corti G. (2008). Acute viral hepatitis increases liver stiffness values measured by transient elastography. *Hepatology*.

[21] Fung J., Lai C.-L., Chan S.-C. (2010). Correlation of liver stiffness and histological features in healthy persons and in patients with occult hepatitis B, chronic active hepatitis B, or hepatitis B cirrhosis. *The American Journal of Gastroenterology*.

[22] Fung J., Lai C.-L., Cheng C., Wu R., Wong D. K.-H., Yuen M.-F. (2011). Mild-to-moderate elevation of alanine aminotransferase increases liver stiffness measurement by transient elastography in patients with chronic hepatitis B. *The American Journal of Gastroenterology*.

[23] Vispo E., Barreiro P., del Valle J. (2009). Overestimation of liver fibrosis staging using transient elastography in patients with chronic hepatitis C and significant liver inflammation. *Antiviral Therapy*.

[24] Millonig G., Reimann F. M., Friedrich S. (2008). Extrahepatic cholestasis increases liver stiffness (fibroScan) irrespective of fibrosis. *Hepatology*.

[25] Trifan A., Sfarti C., Cojocariu C. (2011). Increased liver stiffness in extrahepatic cholestasis caused by choledocholithiasis. *Hepatitis Monthly*.

[26] Colli A., Pozzoni P., Berzuini A. (2010). Decompensated chronic heart failure: Increased liver stiffness measured by means of transient elastography. *Radiology*.

[27] Hopper I., Kemp W., Porapakkham P. (2012). Impact of heart failure and changes to volume status on liver stiffness: non-invasive assessment using transient elastography. *European Journal of Heart Failure*.

[28] Mederacke I., Wursthorn K., Kirschner J. (2009). Food intake increases liver stiffness in patients with chronic or resolved hepatitis C virus infection. *Liver International*.

[29] Arena U., Platon M. L., Stasi C. (2013). Liver stiffness is influenced by a standardized meal in patients with chronic hepatitis C virus at different stages of fibrotic evolution. *Hepatology*.

[30] Petta S., Maida M., Macaluso F. S. (2015). The severity of steatosis influences liver stiffness measurement in patients with nonalcoholic fatty liver disease. *Hepatology*.

[31] Macaluso F. S., Maida M., Cammà C. (2014). Steatosis affects the performance of liver stiffness measurement for fibrosis assessment in patients with genotype 1 chronic hepatitis C. *Journal of Hepatology*.

[32] Perazzo H., Fernandes F. F., Soares J. C. (2015). Learning curve and intra/interobserver agreement of transient elastography in chronic hepatitis C patients with or without HIV co-infection. *Clinics and Research in Hepatology and Gastroenterology*.

[33] Fraquelli M., Rigamonti C., Casazza G. (2007). Reproducibility of transient elastography in the evaluation of liver fibrosis in patients with chronic liver disease. *Gut*.

[34] Boursier J., Konate A., Guilluy M. (2008). Learning curve and interobserver reproducibility evaluation of liver stiffness measurement by transient elastography. *European Journal of Gastroenterology and Hepatology*.

[35] Roca B., Resino E., Torres V., Herrero E., Penades M. (2012). Interobserver discrepancy in liver fibrosis using transient elastography. *Journal of Viral Hepatitis*.

[36] Perazzo H., Fernandes F. F., Gomes A., Terra C., Perez R. M., Figueiredo F. A. F. (2015). Interobserver variability in transient elastography analysis of patients with chronic hepatitis C. *Liver International*.

[37] Nascimbeni F., Lebray P., Fedchuk L. (2015). Significant variations in elastometry measurements made within short-term in patients with chronic liver diseases. *Clinical Gastroenterology and Hepatology*.

[38] Poynard T., Lenaour G., Vaillant J. C. (2012). Liver biopsy analysis has a low level of performance for diagnosis of intermediate stages of fibrosis. *Clinical Gastroenterology and Hepatology*.

[39] European Association for the Study of the Liver (2015). EASL recommendations on treatment of Hepatitis C 2015. *Journal of Hepatology*.

[40] Ingiliz P., Chhay K. P., Munteanu M. (2009). Applicability and variability of liver stiffness measurements according to probe position. *World Journal of Gastroenterology*.

[41] Corpechot C., Gaouar F., El Naggar A. (2014). Baseline values and changes in liver stiffness measured by transient elastography are associated with severity of fibrosis and outcomes of patients with primary sclerosing cholangitis. *Gastroenterology*.

[42] Fernandez M., Trépo E., Degré D. (2015). Transient elastography using Fibroscan is the most reliable noninvasive method for the diagnosis of advanced fibrosis and cirrhosis in alcoholic liver disease. *European Journal of Gastroenterology & Hepatology*.

[43] Poynard T., Vergniol J., Ngo Y. (2014). Staging chronic hepatitis B into seven categories, defining inactive carriers and assessing treatment impact using a fibrosis biomarker (FibroTest) and elastography (FibroScan). *Journal of Hepatology*.

